# Feature Selection via Chaotic Antlion Optimization

**DOI:** 10.1371/journal.pone.0150652

**Published:** 2016-03-10

**Authors:** Hossam M. Zawbaa, E. Emary, Crina Grosan

**Affiliations:** 1 Faculty of Mathematics and Computer Science, Babes-Bolyai University, Cluj-Napoca, Romania; 2 Faculty of Computers and Information, Beni-Suef University, Beni-Suef, Egypt; 3 Faculty of Computers and Information, Cairo University, Cairo, Egypt; 4 Faculty of Computer Studies, Arab Open University, Cairo, Egypt; 5 College of Engineering, Design and Physical Sciences, Brunel University, London, United Kingdom; University of Vermont, UNITED STATES

## Abstract

**Background:**

Selecting a subset of relevant properties from a large set of features that describe a dataset is a challenging machine learning task. In biology, for instance, the advances in the available technologies enable the generation of a very large number of biomarkers that describe the data. Choosing the more informative markers along with performing a high-accuracy classification over the data can be a daunting task, particularly if the data are high dimensional. An often adopted approach is to formulate the feature selection problem as a biobjective optimization problem, with the aim of maximizing the performance of the data analysis model (the quality of the data training fitting) while minimizing the number of features used.

**Results:**

We propose an optimization approach for the feature selection problem that considers a “chaotic” version of the antlion optimizer method, a nature-inspired algorithm that mimics the hunting mechanism of antlions in nature. The balance between exploration of the search space and exploitation of the best solutions is a challenge in multi-objective optimization. The exploration/exploitation rate is controlled by the parameter *I* that limits the random walk range of the ants/prey. This variable is increased iteratively in a quasi-linear manner to decrease the exploration rate as the optimization progresses. The quasi-linear decrease in the variable *I* may lead to immature convergence in some cases and trapping in local minima in other cases. The chaotic system proposed here attempts to improve the tradeoff between exploration and exploitation. The methodology is evaluated using different chaotic maps on a number of feature selection datasets. To ensure generality, we used ten biological datasets, but we also used other types of data from various sources. The results are compared with the particle swarm optimizer and with genetic algorithm variants for feature selection using a set of quality metrics.

## 1 Introduction

The large amounts of data generated today in biology offer more detailed and useful information on the one hand, but on the other hand, it makes the process of analyzing these data more difficult because not all the information is relevant. Selecting the relevant characteristics or attributes of the data is a complex problem. Feature selection (attribute reduction) is a technique for solving classification and regression problems, and it is employed to identify a subset of the features and remove the redundant ones. This mechanism is particularly useful when the number of attributes is large and not all of them are required for describing the data and for further exploring the data attributes in experiments. The basic assumption for employing feature selection is that a large number of features do not necessarily translate into high classification accuracy for many pattern classification problems [1]. Ideally, the selected feature subset will improve the classifier performance and provide a faster and more cost effective classification, which leads to comparable or even better classification or regression accuracy than using all the attributes [2]. In addition, feature selection improves the visualization and the comprehensibility of the induced concepts [3]. Using a tumor as a simple example, there are a large number of attributes that describe it: mitotic activity, tumor invasion, tumor shape and size, vascularization, and growth rate, to name just a few. All of these attributes require measurements and tests that are not always easy to perform. Thus, it will be ideal if the classification of a tumor into benign or malignant (and which stage) could be performed with fewer investigations. The selection of a subset of the features that are relevant enough to perform the classification will be of considerable benefit.

Many studies formulate the feature selection problem as a combinatorial optimization problem, in which the selected feature subset leads to the best data fitting [4]. In real world problems, feature selection is mandatory due to the abundance of noisy, irrelevant or misleading features [5]. These factors can have a negative impact on the classification performance during the learning and operation processes. Two main criteria are used to differentiate the feature selection methods:

1. *Search strategy*: the method employed to generate feature subsets or feature combinations.
2. *Subset quality (fitness)*: the criteria used to judge the quality of a feature subset.

There are two main classes of feature selection methods: wrapper-based methods (apply machine learning algorithms) and filter-based methods (use statistical methods) [6].

The *wrapper-based approach* uses a machine learning technique as part of the evaluation function, which facilitates obtaining better results than the filter-based approach [7], but it has a risk of over-fitting the model and can be computationally expensive, and hence, a very intelligent search method is required to minimize the running time [8]. In contrast, the *filter-based approach* searches for a subset of features that optimize a given data-dependent criterion rather than classification-dependent criteria as in the wrapper methods [1].

In general, the feature selection problem is formulated as a multi-objective problem with two objectives: minimize the size of the selected feature set and maximize the classification accuracy. Typically, these two objectives are contradictory, and the optimal solution is a tradeoff between them.

The size of the search space exponentially increases with respect to the number of features in the dataset [8]. Therefore, an exhaustive search for obtaining the optimal solution is almost impossible in practice. A variety of search techniques have been employed, such as greedy search based on sequential forward selection (SFS) [9] and sequential backward selection (SBS) [10]. However, these feature selection approaches still suffer from stagnation in local optima and expensive computational time [11]. Evolutionary computing (EC) algorithms and other population-based algorithms adaptively search the feature space by employing a set of search agents that communicate in a social manner to reach a global solution [12]. Such methods include genetic algorithms (GAs) [13], particle swarm optimization (PSO) [14], and ant colony optimization (ACO) [3].

GAs and PSO are the most common population-based algorithms. GAs are inspired from the process of evolution via natural selection and survival of the fittest and have the ability to solve complex and non-linear problems; however, in many cases, if no additional mechanisms are employed, they can have poor performance and become trapped in local minima [15]. In PSO, each solution is considered as a particle that is defined by position, fitness, and a speed vector, which defines the moving direction of the particle [16].

The antlion optimization (ALO) algorithm [17] is a relatively recent algorithm that is computationally less expensive than other techniques. The chaotic optimization algorithm (COA) is a global optimization method whose main core contains two phases [18]. The first phase has four steps:

1. Produce a sequence of chaotic points;
2. Map the chaotic points to a sequence of design points in the design space;
3. Compute the fitness (objective function) values based on the design points;
4. Select the point that has the minimum fitness value as the current optimum point.

The second phase has two steps:

1. Assume that the current optimum point is located near the global optimum after a number of iterations;
2. Perform position alteration and search around the current optimum in the descent direction along with the axis directions.

These phases are repeated until a convergence (termination) criterion is met. Chaos is considered to be a deterministic dynamic process and is very responsive to its initial parameters and conditions. The nature of chaos is clearly random and unpredictable, but it also has an element of regularity [18].

The aim of this paper is to enhance the performance of the antlion optimizer for feature selection by using chaos. We are particularly interested in applying our methods to data from biology and medicine, as these data possess a large number of attributes and generally have a small number of instances, which makes the feature selection process more complex.

The remainder of this paper is organized as follows. Subsection 1.1 surveys the existing related work. Section 2 provides background information about the antlion optimization algorithm and chaotic maps. The proposed chaotic version of the antlion optimization (CALO) is presented in Subsection 2.3. The experimental results with discussions are reported in Section 3. The conclusions of this research and directions for future work are presented in Section 4.

### 1.1 Related work

Nature-inspired heuristics, such as genetic algorithms, genetic programming, ant colony optimization, and particle swarm optimization, have been successfully used for feature selection. GA uses the accuracy of classification as a fitness (objective) function and removes or adds a feature according to the ranking information. A feature selection algorithm based on GA using a fuzzy set as the fitness function has been proposed in [19]. PSO with the same fitness function achieves better performance than the GA algorithm in [20]. A multi-objective algorithm for feature selection based on genetic programming has been proposed in [21].

An ACO-based wrapper feature selection algorithm has been applied in network intrusion detection [22]. ACO uses the Fisher discrimination rate to adopt the heuristic information. A feature selection method based on ACO and rough set theory has been proposed in [23]. Logistic map is one of the techniques used by the chaotic behavior and has bounded unstable dynamic behavior. The system proposed in [24] uses the K-nearest neighbor (KNN) classifier with leave-one-out cross-validation (LOOCV) and evaluates the classification performance.

The chaos genetic feature selection optimization method (CGFSO) is proposed in [18]. The method proposed in [25] for text categorization consists of some primary stages, such as feature extraction and feature selection. In the feature selection stage, the method applies feature selection algorithms to obtain a feature subset that can increase the classification accuracy and method performance and can reduce the learning complexity. CGFSO explores the search space with all possible combinations of a given dataset. In addition, each individual in the population represents a candidate solution, with the size of the feature subset being the same as the length of a chromosome [26].

Chaotic time series with the EPNet algorithm is proposed in [27]. The authors present four different methods derived from the classical EPNet algorithm applied in three different chaotic series (Logistic, Lorenz, and Mackey-Glass). The tournament EPNet algorithm obtains the best results for all time series considered, and the network architectures remains of a comparatively limited size. The chaotic time series predictor requires a small network architecture, whereas the addition of neural components may degrade the performance during evolution and consequently provide more survival probabilities to smaller networks in the population [28].

## 2 Methods

### 2.1 Antlion optimization (ALO)

Antlion optimization (ALO) is a bio-inspired optimization algorithm proposed by Mirjalili [17]. The ALO algorithm mimics the hunting mechanism of antlions in nature. Antlions (doodlebugs) belong to the Myrmeleontidae family and Neuroptera order [17]. They primarily hunt in the larvae stage, and the adulthood period is for reproduction. An antlion larvae digs a cone-shaped hole in the sand by moving along a circular path and throwing out sand with its huge jaw. After digging the trap, the larvae hides underneath the bottom of the cone and waits for insects/ants to become trapped in the hole. Once the antlion realizes that a prey is in the trap, it attempts to catch the prey. However, insects are typically not caught immediately and attempt to escape from the trap.

In this case, antlions intelligently throw sand toward the edge of the hole to cause the prey to slide to the bottom of the hole. When a prey is caught in the jaw of an antlion, it is pulled under the soil and consumed. After consuming the prey, antlions throw the leftovers outside the hole and prepare the hole for the next hunt.

#### Artificial antlion

Based on the above description of antlions, Mirjalili uses the following facts and assumptions in the artificial antlion optimization algorithm [17]:

- Prey (ants) move around the search space using different random walks;
- Random walks are affected by the traps of antlions;
- Antlions can build holes proportional to their fitness (the higher the fitness, the larger the hole);
- Antlions with larger holes have a higher probability of catching ants;
- Each ant can be caught by an antlion in each iteration;
- The range of random walks is decreased adaptively to simulate sliding ants toward antlions;
- If an ant becomes fitter than an antlion, this means that the ant is caught and pulled under the sand by the antlion;
- An antlion repositions to the most recently caught prey and builds a hole to improve its chance of catching another prey after each hunt.

Formally, the antlion optimization algorithm is given in Algorithm 1.

**Algorithm 1:** Antlion optimization (ALO) algorithm

**Input:** Search space, fitness function, numbers of ants and antlions, number of iterations *T*

**Output:** The elitist antlion and its fitness

  1. Randomly initialize a population of ant positions *Ant* and a population of antlion positions *Antlion*.

  2. Calculate the fitness of all the ants and antlions.

  3. Find the fittest antlion; Elite.

  4. *t* = 0.

  5. **while** (*t* ≤ *T*)

   **foreach** *Ant*_*i*_ **do**

    **• Select an antlion using Roulette wheel.**

    **• Slide ants toward the antlion as in Eq (2).**

    **• Create a random walk for the *Ant*_*i*_ and normalize it, as shown in Eqs (4) and (5) for modeling trapping, Eq (6) for random walk, and Eq (8) for walk normalization.**

   **end**

  **6. Calculate the fitness of all ants.**

  **7. Replace an antlion with its corresponding ant if the ant becomes fitter following Eq (1).**

  **8. Update the elite if an antlion becomes fitter than the current elite.**

  **9.** *t* = *t*+**1**

   **end while**

The antlion optimizer applies the following steps to an individual *antlion*:

1. ***Building a trap:*** a roulette wheel is used to model the hunting capability of antlions. Ants are assumed to be trapped in only one *selected* antlion hole. The ALO algorithm requires a roulette wheel operator for selecting antlions based on their fitness during optimization. This mechanism provides high chances to the fitter antlions for catching prey or ants.
2. ***Catching prey and re-building the hole:*** this is the final stage in hunting, in which the antlion consumes the ant. It is assumed that prey catching occurs when the ant becomes fitter (goes inside sand) than its corresponding antlion. The antlion has to update his position to the latest position of the hunted ant to increase its chance of catching new prey. Eq (1) reflects this process:
  $$\begin{array}{c} Antlion_{j}^{t}=Ant_{i}^{t}\;\text{If}\;f(Ant_{i}^{t})\;\text{is}\;\text{better}\;\text{than}\;f(Antlion_{j}^{t}), \end{array}$$ (1)
  where:
  - *t* shows the current iteration;
  - $Antlion_{j}^{t}$ shows the position of the antlion *j* at iteration *t*;
  - $Ant_{i}^{t}$ indicates the position of the ant *i* at iteration *t*.

The antlion optimizer applies the following four operations to an individual *ant*:

1. ***Sliding ants toward antlion:*** antlions shoot sand toward the center of the hole once they realize that an ant is in the trap. This behavior causes the trapped ant that is attempting to escape to slide down. To mathematically model this behavior, the radius of the ants’ random walk hyper-sphere is *decreased* adaptively using Eqs (2) and (3).
  $$\begin{array}{c} c^{t}=\frac{c^{t}}{I}, \end{array}$$ (2)
  where:
  - *c*^*t*^ is the minimum of all variables at iteration *t*;
  - *I* is a ratio, which is defined in Eq (3):
  $$\begin{array}{c} I=10^{w}\frac{t}{T}, \end{array}$$ (3)
  where:
  - *t* is the current iteration;
  - *T* is the maximum number of iterations;
  - *w* is a constant defined based on the current iteration (*w* = 2 when *t* > 0.1*T*, *w* = 3 when *t* > 0.5*T*, *w* = 4 when *t* > 0.75*T*, *w* = 5 when *t* > 0.9*T*, and *w* = 6 when *t* > 0.95*T*). Basically, the constant *w* can adjust the accuracy level of exploitation.
2. ***Trapping in the antlion holes:*** by modeling the sliding of prey toward the antlion, the ant is trapped in the antlion’s hole. In other words, the walk of the ant becomes bounded by the position of the antlion, which can be modeled by changing the range of the ant random walk toward the antlion position as in Eqs (4) and (5):
  $$\begin{array}{c} c_{i}^{t}=c^{t}+Antlion_{j}^{t}, \end{array}$$ (4)
  $$\begin{array}{c} d_{i}^{t}=d^{t}+Antlion_{j}^{t}, \end{array}$$ (5)
  where:
  - *c*^*t*^ is the minimum of all variables at iteration *t*;
  - *d*^*t*^ is the maximum of all variables at iteration *t*;
  - $c_{i}^{t}$ is the minimum of all variables for ant *i*;
  - $d_{i}^{t}$ is the maximum of all variables for ant *i*;
  - $Antlion_{j}^{t}$ represents the position of the antlion *j* at iteration *t*.
3. ***Random walks of ants:*** Random walks are based on Eq (6):
  $$\begin{array}{c} X\left(t\right)=[0,cumsum\left(2r\left(t_{1}\right)-1\right);cumsum\left(2r\left(t_{2}\right)-1\right);\\ ...;cumsum(2r\left(t_{T}\right)-1)], \end{array}$$ (6)
  where:
  - *cumsum* calculates the cumulative sum;
  - *T* is the maximum number of iterations;
  - *t* is the step of the random walk (iteration);
  - *r*(*t*) is a stochastic function defined as:
  $$\begin{array}{c} r\left(t\right)=\left\{\begin{array}{c} 1\;\text{if}\;rand>0.5\\ 0\;\text{if}\;rand\leq 0.5, \end{array}\right. \end{array}$$ (7)
  where *rand* is a random number generated with a uniform distribution over [0, 1].
  To keep the random walks inside the search space, they are normalized using Eq (8) (min–max normalization):
  $$\begin{array}{c} X_{i}^{t}=\frac{\left(X_{i}^{t}-a_{i}\right)\times \left(d_{i}-c_{i}^{t}\right)}{\left(b_{i}^{t}-a_{i}\right)}+c_{i}, \end{array}$$ (8)
  where:
  - *a*_*i*_ is the minimum random walk for variable *i*;
  - *b*_*i*_ is the maximum random walk for variable *i*;
  - $c_{i}^{t}$ is the minimum of variable *i* at iteration *t*;
  - $d_{i}^{t}$ is the maximum of variable *i* at iteration *i*.
4. ***Elitism:*** to maintain the best solution(s) across iterations, elitism has to be applied. In this work, we consider that the random walk of an ant is guided by the selected antlion and by the elite antlion, and hence, the repositioning of a given ant follows the average of both random walks, as shown in Eq (9):
  $$\begin{array}{c} Ant_{i}^{t}=\frac{R_{A}^{t}+R_{E}^{t}}{2}, \end{array}$$ (9)
  where:
  - $R_{A}^{t}$ is the random walk around the antlion selected using a roulette wheel;
  - $R_{E}^{t}$ is the random walk around the elite antlion.

### 2.2 Chaotic maps

Chaos means a condition or place of great disorder or confusion [29]. Chaotic systems are deterministic systems that exhibit irregular (or even random) behavior and a sensitive dependence on the initial conditions. Chaos is one of the most popular phenomena that exist in nonlinear systems, whose action is complex and similar to that of randomness [30]. Chaos theory studies the behavior of systems that follow deterministic laws but appear random and unpredictable, i.e., dynamical systems. To be referred to as chaotic, the dynamical system must satisfy the following chaotic properties [29]:

1. sensitive to initial conditions;
2. topologically mixing;
3. dense periodic orbits;
4. ergodic;
5. stochastically intrinsic.

Chaotic variables can go through all states in certain ranges according to their own regularity without repetition [30]. Due to the ergodic and dynamic properties of chaos variables, chaos search is more capable of hill-climbing and escaping from local optima than random search, and thus, it has been applied for optimization [30]. It is widely recognized that chaos is a fundamental mode of motion underlying almost all natural phenomena. A chaotic map is a map that exhibits some type of chaotic behavior [29]. The common chaotic maps in the literature are as follows:

1. ***Logistic map:*** this map is one of the simplest chaotic maps [31], as defined in Eq (10):
  $$\begin{array}{c} x_{k+1}=ax_{k}\left(1-x_{k}\right), \end{array}$$ (10)
  where:
  - *x*_*k*_ ∈ (0, 1) under the condition that *x*_0_ ∈ [0, 1], 0 < *a* ≤ 4;
  - *k* is the iteration number.
2. ***Sinusoidal map:*** represented by Eq (11) [31]:
  $$\begin{array}{c} x_{k+1}=ax_{k}^{2}sin\left(\pi x_{k}\right), \end{array}$$ (11)
  which generates chaotic numbers in the range (0, 1) with *a* = 2.3.
3. ***Tent map:*** resembles the logistic map due to its topologically conjugate behavior. A tent map can display a range of dynamical behaviors from predictable to chaotic depending on the value of its multiplier, as shown in Eqs (12) and (13):
  $$\begin{array}{c} x_{k+1}=G\left(x_{k}\right), \end{array}$$ (12)
  $$\begin{array}{c} G\left(x\right)=\left\{\begin{array}{cc} \frac{x}{0.7}, & x<0.7\\ \frac{1}{0.3}x\left(1-x\right) & otherwise \end{array}\right. \end{array}$$ (13)
4. ***Singer map:*** given in Eq (14) [32]:
  $$\begin{array}{c} x_{k+1}=\mu \left(7.86x_{k}-23.31x_{k}^{2}+28.75x_{k}^{3}-13.3x_{k}^{4}\right), \end{array}$$ (14)
  with *x*_*k*_ ∈ (0, 1) under the condition that *x*_0_ ∈ (0, 1), *μ* ∈ [0.9, 1.08].
5. ***Piecewise map:*** given in Eq (15) [33]:
  $$\begin{array}{c} x_{k+1}=\left\{\begin{array}{cc} \frac{x}{p}, & 0\leq x_{k}<p\\ \frac{x_{k}-p}{0.5-p} & p\leq x_{k}<0.5\\ \frac{1-p-x_{k}}{0.5-p} & 0.5\leq x_{k}<\left(1-p\right)\\ \frac{1-x_{k}}{p} & \left(1-p\right)\leq x_{k}<1 \end{array}\right. \end{array}$$ (15)
  where *p* is a constant defined between 0 and 1.

### 2.3 The novel chaotic antlion optimization (CALO)

In this section, we present our chaotic antlion optimization (CALO) algorithm based on k-nearest neighbor (KNN) for feature selection. *Exploration* can be defined as the acquisition of new information through searching [34]. Exploration is a main concern for all optimizers because it might lead to new search regions that might contain better solutions. *Exploitation* is defined as the application of known information. The good sites are exploited via the application of a local search. The selection process should be balanced between random selection and greedy selection to bias the search toward fitter candidate solutions (*exploitation*) while promoting useful diversity into the population (*exploration*) [34].

**Fig 1:**
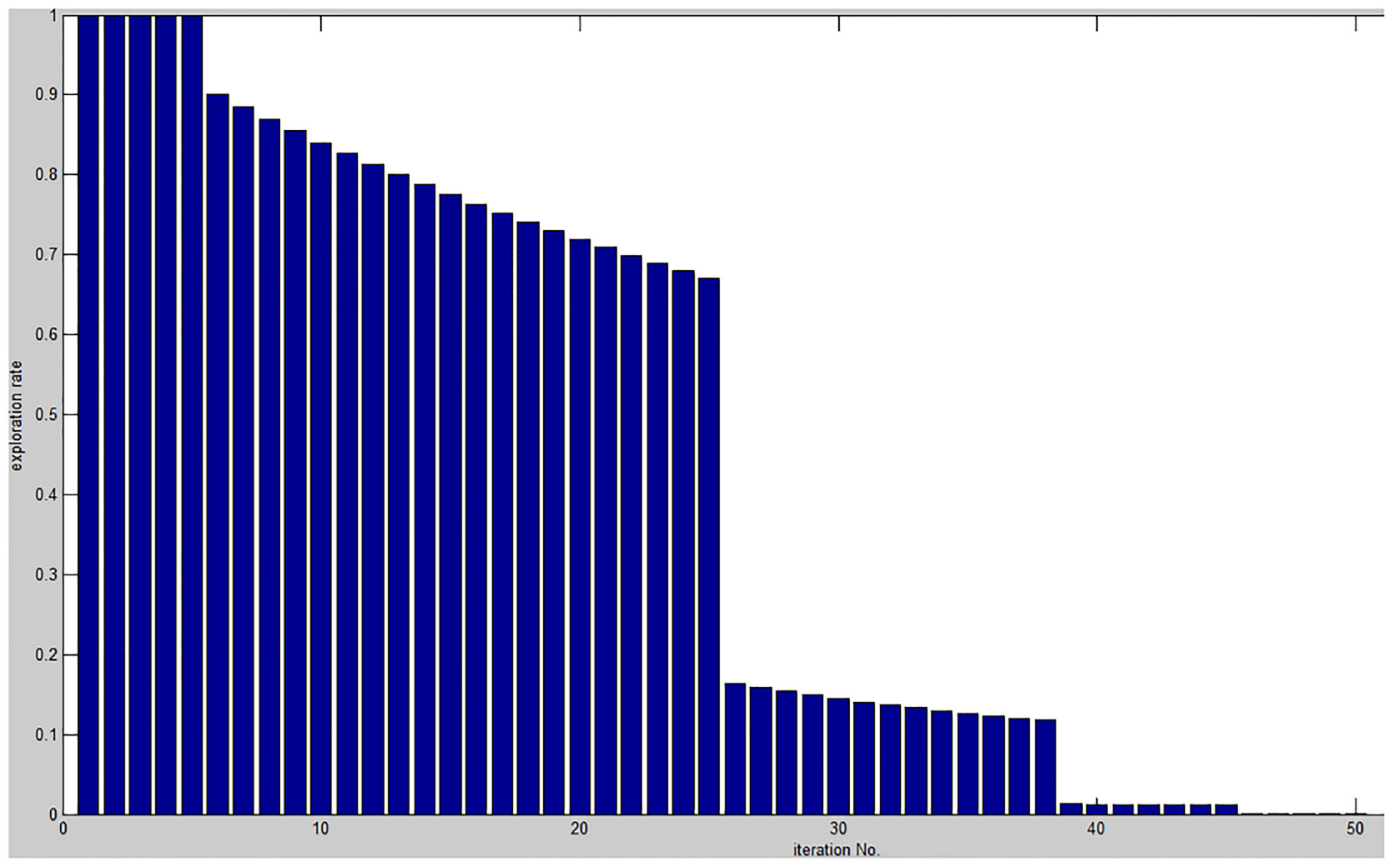
Exploration rate ($\frac{1}{I}$) at different iterations in the original ALO.

Parameter *I* controls the trade-off between exploration and exploitation in the original antlion optimization algorithm. This parameter is linearly decreased to allow more exploration at the beginning of the optimization process, while exploitation becomes more important at the end of the optimization. Therefore, half of the optimization resources are consumed in exploration, whereas the remaining time is dedicated to exploitation, as shown in (Fig 1).

Although the algorithm proved efficient for solving numerous optimization problems, it still possesses the following drawbacks:

1. *Sub-optimal selection:* at the beginning of the optimization process, *I* is small, which makes the random walk unbounded in the search space and allows an ant to apply random walk in almost the entire search space. This may cause the algorithm to select sub-optimal solutions.
2. *Stagnation:* once the algorithm approaches the end of the optimization process, it becomes difficult to escape local optima and find better solutions because its exploration capability is very limited; *I* becomes very large, thereby limiting the boundaries for the random walk. This causes the algorithm to continue enhancing solutions that have already been found, even if they are sub-optimal.

**Fig 2:**
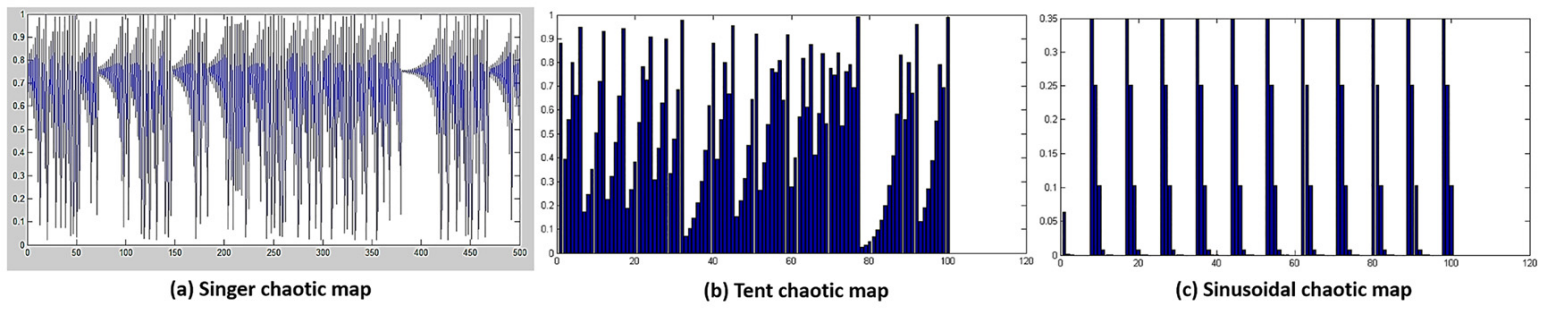
The exploration and exploitation values of different chaotic maps.

These problems motivate our work on adapting $\frac{1}{I}$ to obtain successive periods of exploration and exploitation. Therefore, when reaching a solution, exploitation will be applied, followed by another exploration, which may jump to another promising area, followed by using exploitation again to further enhance the solution found, and so on. Chaotic systems with their interesting properties, such as topologically mixing and dense periodic orbits, ergodicity and intrinsic stochasticity, can be used to adapt this parameter, allowing for the required mix between exploration and exploitation. (Fig 2a) presents an example of a chaos map for the values of *I* for 500 iterations, in which we can observe alternating regions of exploration and exploitation. A small variation in the period means exploitation, whereas a larger variation in the period means exploration. The *tent* map smoothly and periodically decrements the exploration rate, while the sinusoidal map abruptly switches between exploration and exploitation, which may cause loss of the optimal solution and lead to worse performance (as shown in (Fig 2b and 2c).

**Fig 3:**
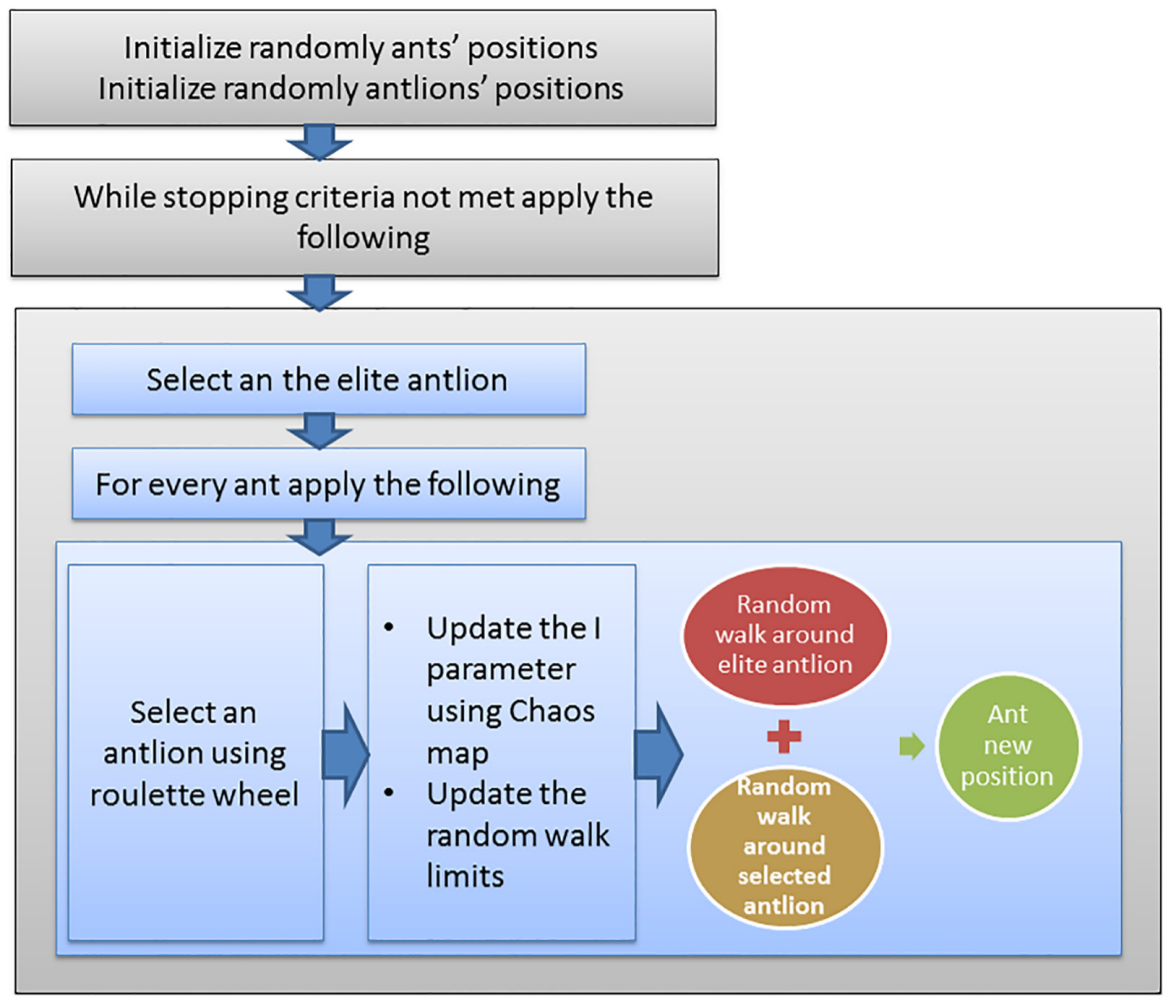
The proposed chaotic antlion optimization (CALO).

The proposed CALO algorithm is schematically presented in (Fig 3). The search strategy of the wrapper-based approach explores the feature space to find a feature subset guided by the classification performance of individual feature subsets.

This approach may be slow because the classifier must be retrained on all the candidate subsets of the feature set and its performance must be measured. Therefore, an intelligent search of the feature space is required. The goals are to maximize the classification performance *P* and to minimize the number of selected features *N*_*f*_. The fitness function is given in Eq (16) [35]:

$$\begin{array}{c} minimize\;\alpha \left(1-P\right)+\left(1-\alpha \right)\left(\frac{N_{f}}{N_{t}}\right), \end{array}$$ (16)

where:

- *N*_*f*_ is the size of the selected feature subset;
- *N*_*t*_ is the total number of features in the dataset;
- *α* ∈ [0, 1] defines the weights of the sub-goals;
- *P* is the classification performance measured as in Eq (17).

$$\begin{array}{c} P=\frac{N_{c}}{N}, \end{array}$$ (17)

where *N*_*c*_ is the number of correctly classified data instances and *N* is the total number of instances in the dataset.

The number of dimensions in the optimization is the same as the number of features, with each feature related to a dimension and each variable limited to the range [0, 1]. To determine whether a feature will be selected at the evaluation stage, a static threshold of 0.5 is used, as shown in Eq (18):

$$\begin{array}{c} y_{ij}=\left\{\begin{array}{c} 0\;\text{If(}x_{ij}<0.5\text{)}\\ 1\;\text{Otherwise} \end{array}\right. \end{array}$$ (18)

where *y*_*ij*_ is the discrete representation of solution vector *x*, and *x*_*ij*_ *x*_*ij*_ is the continuous position of the search agent *i* in dimension *j*.

## 3 Results and Discussion

### 3.1 Experimental setup

#### Datasets

Table 1 summarizes the 18 datasets used for the experiments. The datasets are taken from the UCI data repository [36]. We use ten biological datasets to validate the performance of our method and its potential applicability for data generated in biology. In addition, we use eight datasets from other areas to show the general adaptability of our method. Each dataset is divided into 3 equal parts for *training*, *validation*, and *testing*. The *training* set is used to train a classifier through optimization and at the final evaluation. The *validation* set is used to assess the performance of the classifier at the optimization time. The *testing* set is used to evaluate the selected features.

**Table 1:** Datasets used in the experiments.

Dataset	No. of features	No. of samples	Scientific area
Breastcancer	9	699	Biology
BreastEW	30	569	
Exactly [37]	13	1000	
Exactly2 [37]	13	1000	
HeartEW	13	270	
Lymphography	18	148	
M-of-n	13	1000	
PenglungEW	325	73	
SonarEW	60	208	
SpectEW	22	267	
CongressEW	16	435	Politics
IonosphereEW	34	351	Electromagnetic
KrvskpEW	36	3196	Game
Tic-tac-toe	9	958	Game
Vote	16	300	Politics
WaveformEW	40	5000	Physics
WineEW	13	178	Chemistry
Zoo	16	101	Artificial, 7 classes of animals

Four different optimization methods are compared in this study: CALO with five different chaotic maps—logistic, singer, tent, piecewise, and sinusoidal; the original ALO; particle swarm optimization [14]; and genetic algorithms [13]. The parameter settings for all the algorithms are presented in Table 2.

**Table 2:** Parameter settings for CALO.

Parameter	Value
No of search agents	8
No of iterations	70
Problem dimension	Same as number of features in any given database
Search domain	[0 1]

### 3.2 Performance metrics

Each algorithm has been applied 20 times with random positioning of the search agents except for the full features selected solution, which was forced to be a position for one of the search agents. Forcing the full features solution guarantees that all subsequent feature subsets, if selected as the global best solution, are fitter than it. The well-known KNN is used as a classifier to evaluate the final classification performance for individual algorithms with k = 5 [1]. Repeated runs of the optimization algorithms were used to test their convergence capability. The indicators (measures) used to compare the different algorithms are as follows:

- *Statistical mean:* is the average performance of a stochastic optimization algorithm applied *M* times and is given in Eq (19):
  $$\begin{array}{c} Mean=\frac{1}{M}\sum_{i=1}^{M}g_{*}^{i}, \end{array}$$ (19)
  where $g_{*}^{i}$ is the optimal solution that resulted at the *i*−*th* application of the algorithm.
- *Statistical best:* is the minimum fitness function value (or best value) obtained by an optimization algorithm in *M* independent applications, as shown in Eq (20):
  $$\begin{array}{c} Best=min_{i=1}^{M}g_{*}^{i}, \end{array}$$ (20)
- *Statistical worst:* is the maximum fitness function value (or worst value) obtained by an optimization algorithm in *M* independent applications, as in Eq (21):
  $$\begin{array}{c} Worst=max_{i=1}^{M}g_{*}^{i}, \end{array}$$ (21)
- *Statistical standard deviation (std):* is used as an indicator of the optimizer stability and robustness: when *Std* is small, the optimizer always converges to the same solution, whereas large values of *std* represent close to random results, as shown in Eq (22):
  $$\begin{array}{c} Std=\sqrt{\frac{1}{M-1}\sum {\left(g_{*}^{i}-Mean\right)}^{2}}, \end{array}$$ (22)
- *Average classification accuracy:* describes how accurate the classifier is given the selected feature set, as shown in Eq (23).
  $$\begin{array}{c} Avg\_Perf=\frac{1}{M}\sum_{j=1}^{M}\frac{1}{N}\sum_{i=1}^{N}Match\left(C_{i},L_{i}\right), \end{array}$$ (23)
  where:
  - *N* is the number of instances in the test set;
  - *C*_*i*_ is the classifier output label for data instance *i*;
  - *L*_*i*_ is the reference class label for data instance *i*;
  - *Match* is a function that outputs 1 when the two input labels are the same and outputs 0 otherwise.
- *Average selection size (reduction):* represents the fraction of selected features from all feature sets, as shown in Eq (24).
  $$\begin{array}{c} Avg\_Selection\_Size=\frac{1}{M}\sum_{i=1}^{M}\frac{size(g_{*}^{i})}{N_{t}}, \end{array}$$ (24)
  where *N*_*t*_ is the number of features in the original dataset.
- *Average fisher score (f-score):* is a measure that evaluates a feature subset such that in the data space spanned by the selected features, the distances between data instances in different classes are as large as possible, while the distances between data instances in the same class are as small as possible [4]. *F-score* in this work is calculated for individual features given the class labels and for *M* independent applications of an algorithm, as given in Eq (25):
  $$\begin{array}{c} F_{j}=\frac{\sum_{k=1}^{c}n_{k}{\left(\mu_{k}^{j}-\mu^{j}\right)}^{2}}{{\left(\sigma^{j}\right)}^{2}}, \end{array}$$ (25)
  where:
  - *F*_*j*_ is the fisher score for feature *j*;
  - *μ*^*j*^ is the mean of the entire dataset;
  - (*σ*^*j*^)^2^ is the standard deviation of the entire dataset;
  - *n*_*k*_ is the size of class *k*;
  - $\mu_{k}^{j}$ is the mean of class *k*.

**Algorithms used for comparison:** our comparisons include the following algorithms:

- ALO: the original antlion optimization
- CALO-Log: chaotic ALO with logistic map
- CALO-Piec: chaotic ALO with piece-wise chaotic map
- CALO-Singer: chaotic ALO with singer chaotic map
- CALO-Sinu: chaotic ALO with sinusoidal map
- CALO-Tent: chaotic ALO with tent map
- GA: genetic algorithm
- PSO: particle swarm optimization.

### 3.3 Analysis and discussion

**Fig 4:**
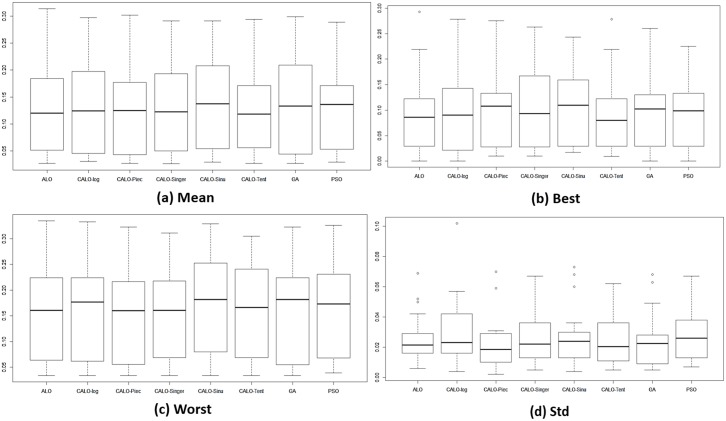
The fitness values obtained from different methods for all 18 datasets.

**Fig 5:**
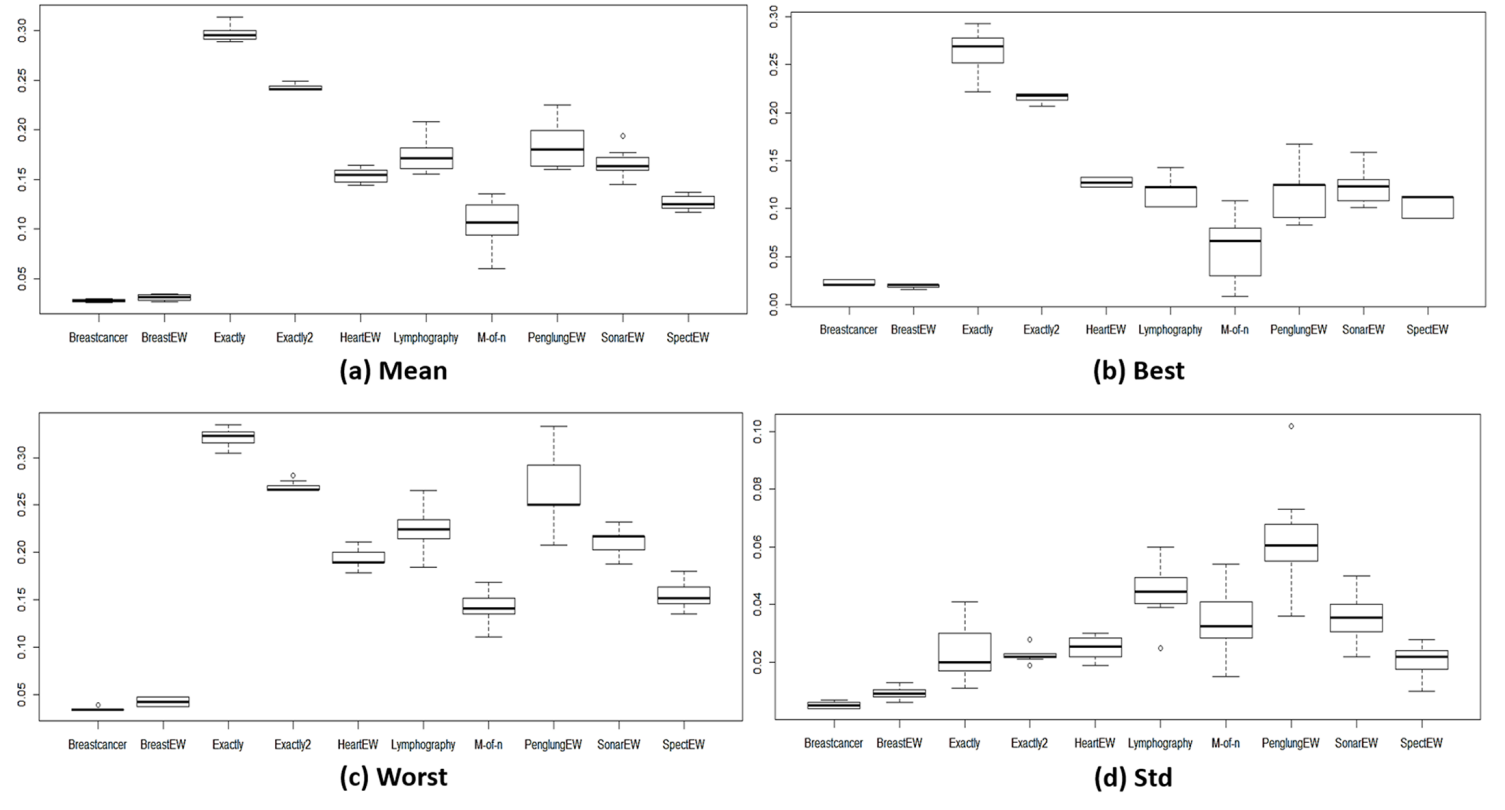
The fitness values obtained from different methods for biological datasets.

**Fig 6:**
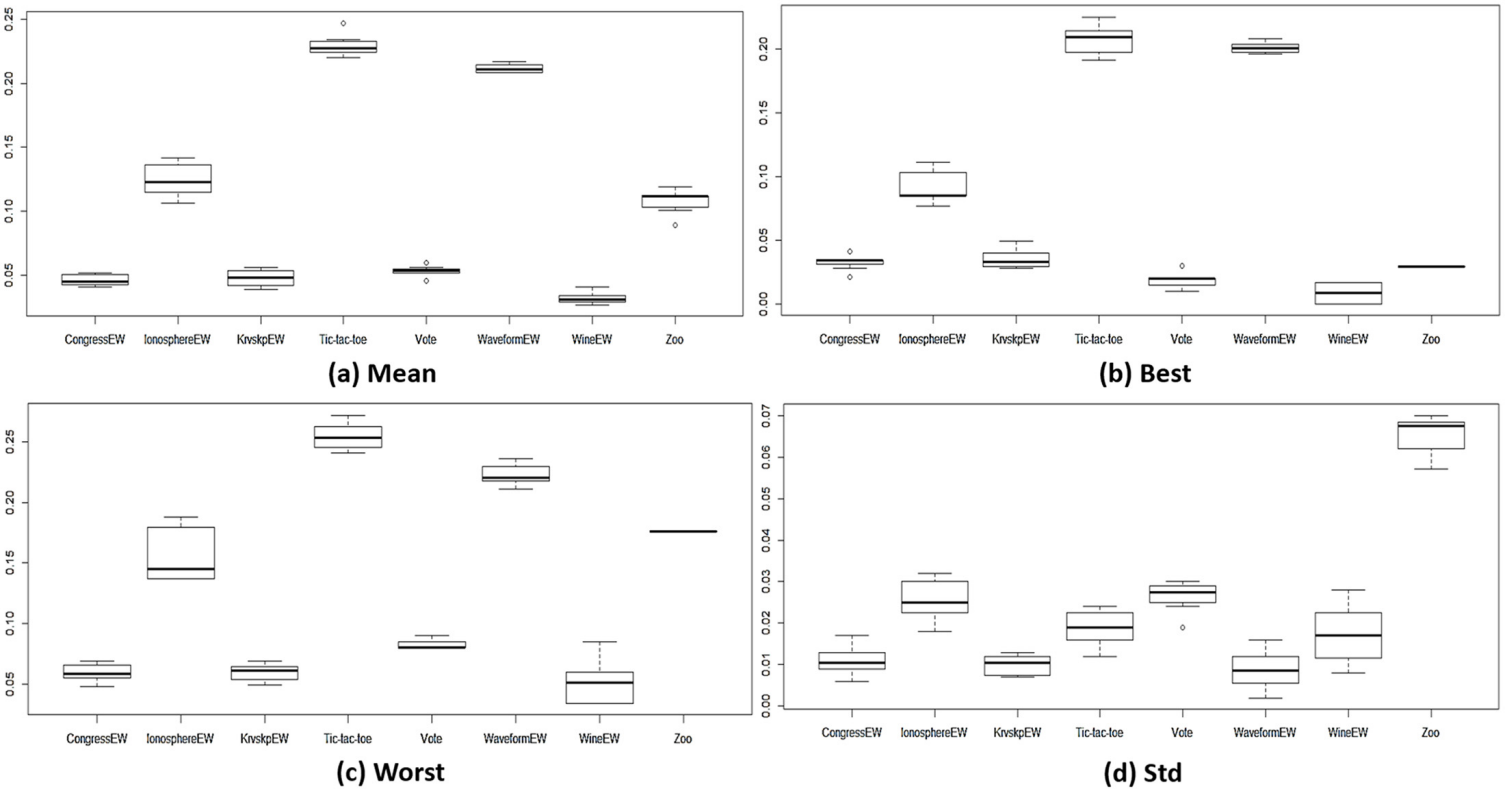
The fitness values obtained from different methods for non-biological datasets.

Fig 4 shows the average statistical mean fitness, best fitness, worst fitness, and the standard deviation for all the methods used and for all 18 datasets. The results for the biological datasets are presented in (Fig 5), and those for the other non-biological datasets are presented in (Fig 6). We can observe that ALO and CALO generally perform better than GA and PSO. The search method adopted in ALO is more explorative than the one used in GA and PSO because ALO performs a local search around a roulette wheel selected solution, and in this way, other areas (apart from the area around the current best) are explored. Because of the balanced control of exploration and exploitation, the CALO algorithm outperforms the original ALO. The nonsystematic adaptation of exploration rate in the CALO allows the successive local and global searching and helps escaping from local minima that commonly exist in the search space. The *tent* chaos map outperforms the other chaos maps, whereas the *sinusoidal* map provides the worst chaotic result.

To assess the stability of the stochastic algorithms and the stability to converge to the same optimal solution, we measure the statistical standard deviation (std) of the fitness values over different runs. The minimum for the std measure is obtained by CALO in almost all the datasets, which reaffirms that CALO is more stable and can converge to the same optimal solution regardless of its stochastic and chaotic manner. In addition, we can see that the *tent* map still performs better than the other maps in terms of its repeatability. The results for the classification accuracy presented in Table 3 show that CALO obtains the best results for 11 of the datasets, thus demonstrating the capability of CALO to find optimal feature combinations ensuring good test performance.

**Table 3:** Average classification accuracy of 20 independent runs for GA, PSO, ALO, and 5 different chaos maps of CALO.

Dataset	ALO	CALO-Log	CALO-Piec	CALO-Singer	CALO-Sinu	CALO-Tent	GA	PSO
**Breastcancer**	0.954	0.955	0.955	0.950	**0.957**	0.951	0.955	0.951
**BreastEW**	0.943	0.943	0.946	0.949	0.948	**0.952**	0.946	0.942
**Exactly**	0.660	0.675	0.655	0.671	**0.681**	0.661	0.659	0.671
**Exactly2**	0.741	0.737	0.747	0.730	**0.761**	0.736	0.728	0.737
**HeartEW**	**0.824**	0.813	0.813	0.811	0.822	0.820	0.802	**0.824**
**Lymphography**	0.744	**0.772**	0.732	0.728	0.720	0.724	0.744	0.692
**M-of-n**	0.875	0.891	0.882	0.880	0.851	**0.930**	0.867	0.841
**PenglungEW**	0.658	0.659	0.625	0.644	0.603	0.632	**0.676**	0.627
**SonarEW**	0.714	0.723	0.680	0.734	0.697	0.717	0.723	**0.737**
**SpectEW**	0.787	0.784	0.780	0.778	0.778	0.787	0.782	**0.800**
**CongressEW**	0.946	0.941	0.942	0.950	0.942	0.932	0.938	**0.960**
**IonosphereEW**	0.848	0.819	0.838	**0.853**	0.836	0.843	0.824	0.815
**KrvskpEW**	0.938	0.948	0.953	**0.953**	0.941	0.930	0.950	0.946
**Tic-tac-toe**	**0.766**	0.737	0.739	0.754	0.725	0.732	0.739	0.722
**Vote**	0.914	**0.928**	0.922	0.920	0.918	0.882	0.914	0.898
**WaveformEW**	**0.769**	0.765	0.767	0.766	0.764	0.762	0.766	0.757
**WineEW**	0.937	0.930	0.950	0.950	0.953	**0.957**	0.947	0.923
**Zoo**	0.836	0.798	0.805	0.854	0.846	**0.860**	0.824	0.805

Table 4 summarizes the results for the size of the selected feature subsets. We can see that CALO, while outperforming all the other methods in terms of classification performance, has comparable values with the other approaches for the number of features selected.

**Table 4:** Average selection size of 20 independent runs for GA, PSO, ALO, and 5 different chaos maps of CALO.

Dataset	ALO	CALO-Log	CALO-Piec	CALO-Singer	CALO-Sinu	CALO-Tent	GA	PSO
**Breastcancer**	0.800	0.844	0.756	0.689	0.822	0.733	0.667	**0.600**
**BreastEW**	0.567	0.753	0.580	0.687	0.653	0.680	0.527	**0.460**
**Exactly**	0.538	0.538	0.723	0.692	0.754	0.738	**0.508**	0.615
**Exactly2**	0.785	0.492	0.646	0.585	0.477	0.738	0.508	**0.415**
**HeartEW**	0.677	**0.538**	0.615	0.738	0.662	0.723	0.615	0.677
**Lymphography**	**0.400**	0.456	0.456	0.600	0.611	0.456	0.522	0.433
**M-of-n**	0.815	0.800	0.754	0.846	0.954	0.631	0.708	**0.492**
**PenglungEW**	0.266	0.419	0.316	0.357	0.442	**0.259**	0.494	0.476
**SonarEW**	0.357	0.350	0.433	0.537	0.360	**0.210**	0.483	0.497
**SpectEW**	0.391	0.418	**0.382**	0.400	0.427	0.527	0.518	0.455
**CongressEW**	**0.300**	0.362	0.512	0.388	0.325	0.388	0.450	0.375
**IonosphereEW**	0.324	0.200	0.694	**0.135**	0.524	0.235	0.553	0.535
**KrvskpEW**	0.739	0.611	0.561	0.550	0.672	0.594	0.528	**0.489**
**Tic-tac-toe**	0.711	0.756	0.711	0.756	0.622	0.667	0.711	**0.533**
**Vote**	0.475	0.588	0.613	0.500	**0.313**	0.625	0.325	0.475
**WaveformEW**	0.850	0.765	0.825	0.855	0.805	0.810	**0.575**	0.600
**WineEW**	0.615	0.585	0.462	0.554	0.677	0.615	**0.446**	0.508
**Zoo**	0.637	0.662	0.700	0.613	**0.588**	**0.588**	0.613	0.600

**Table 5:** An example of the feature selection size (reduction) for each optimization algorithm using the *Breastcancer* dataset.

Optimizer	Selected features		
	Total No.	Indices	Labels
**CALO-Tent**	4	1, 2, 5, 6	Clump Thickness, Uniformity of Cell Size, Single Epithelial Cell Size, Bare Nuclei
**CALO-Sinu**	6	1, 3, 4, 6, 7, 9	Clump Thickness, Uniformity of Cell Shape, Marginal Adhesion, Bare Nuclei, Bland Chromatin, Mitoses
**CALO-Singer**	7	1, 2, 3, 5, 6, 7, 9	Clump Thickness, Uniformity of Cell Size, Uniformity of Cell Shape, Single Epithelial Cell Size, Bare Nuclei, Bland Chromatin, Mitoses
**CALO-Piec**	7	1, 2, 3, 5, 6, 7, 9	Clump Thickness, Uniformity of Cell Size, Uniformity of Cell Shape, Single Epithelial Cell Size, Bare Nuclei, Bland Chromatin, Mitoses
**CALO-Log**	5	1, 3, 6, 7, 9	Clump Thickness, Uniformity of Cell Shape, Bare Nuclei, Bland Chromatin, Mitoses
**ALO**	8	1, 2, 4, 5, 6, 7, 8, 9	Clump Thickness, Uniformity of Cell Size, Marginal Adhesion, Single Epithelial Cell Size, Bare Nuclei, Bland Chromatin, Normal Nucleoli, Mitoses
**GA**	5	1, 3, 6, 7, 9	Clump Thickness, Uniformity of Cell Shape, Bare Nuclei, Bland Chromatin, Mitoses
**PSO**	5	1, 3, 5, 6, 7	Clump Thickness, Uniformity of Cell Shape, Single Epithelial Cell Size, Bare Nuclei, Bland Chromatin

**Table 6:** An example of the feature selection size (reduction) for each optimization algorithm using the *HeartEW* dataset.

Optimizer	Selected features		
	Total No.	Indices	Labels
**CALO-Tent**	5	3, 9, 10, 11, 12	chest pain type, exercise-induced angina, oldpeak, slope of the peak exercise ST segment, number of major vessels
**CALO-Sinu**	6	1, 3, 7, 9, 12, 13	age, chest pain type, resting electrocardiographic, exercise-induced angina, number of major vessels, defect type
**CALO-Singer**	8	1, 3, 4, 7, 8, 11, 12, 13	age, chest pain type, resting blood pressure, resting electrocardiographic, maximum heart rate, slope of the peak exercise ST segment, number of major vessels, defect type
**CALO-Piec**	8	1, 2, 3, 6, 8, 10, 11, 12	age, sex, chest pain type, fasting blood sugar, maximum heart rate, oldpeak, slope of the peak exercise ST segment, number of major vessels
**CALO-Log**	6	1, 2, 3, 7, 12, 13	age, sex, chest pain type, resting electrocardiographic, number of major vessels, defect type
**ALO**	6	2, 3, 5, 11, 12, 13	sex, chest pain type, serum cholesterol, slope of the peak exercise ST segment, number of major vessels, defect type
**GA**	7	3, 5, 8, 9, 10, 11, 12	chest pain type, serum cholesterol, maximum heart rate, exercise-induced angina, oldpeak, slope of the peak exercise ST segment, number of major vessels
**PSO**	8	1, 2, 3, 6, 7, 10, 12, 13	age, sex, chest pain type, fasting blood sugar, resting electrocardiographic, oldpeak, number of major vessels, defect type

Tables 5 and 6 show particular feature selection size (reduction) examples for the *Breastcancer* dataset, which has 9 input features, and for the *HeartEW* dataset, which has 13 input features. From the Breastcancer dataset, we can observe that CALO suggests that only four of the features are good enough to classify a tumor. As might be evident, it is particularly preferred in biology and medicine to consider a small number of biomarkers for a disease because this involve fewer experiments, which may sometimes be difficult to perform and have side effects for the patient. For the Heart dataset, our method suggests that five of the data attributes will assure the same precision in performing the classification as if we consider all the features. Such tools could be of real help in the future as they will lead to fewer patient investigations and can lower the costs involved. Overall, while comparing CALO with GA and PSO, we observe that CALO almost always obtains better or very similar classification accuracy with a lower number of features selected. In the majority of the tests performed, on average, approximately 75% of the features selected by CALO are in common with the features selected by GA or PSO, but in most of the cases, the set of features selected by CALO is included in the set of features selected by GA and PSO.

F-score values are given in Table 7, where we can again observe that CALO using the *tent* map obtains the best results overall. Additionally, note that the worst performing map is the *sinusoidal* map.

**Table 7:** Average f-score of 20 independent runs for GA, PSO, ALO, and 5 different chaos maps of CALO.

Dataset	ALO	CALO-Log	CALO-Piec	CALO-Singer	CALO-Sinu	CALO-Tent	GA	PSO
**Breastcancer**	9.159	10.084	8.780	8.251	9.661	8.417	8.155	**7.454**
**BreastEW**	7.855	9.552	7.792	9.085	8.349	9.625	7.382	**6.872**
**Exactly**	0.017	**0.016**	0.022	0.023	0.024	0.025	0.018	0.021
**Exactly2**	0.025	**0.014**	0.020	0.018	**0.014**	0.023	0.015	0.015
**HeartEW**	1.408	**1.241**	1.301	1.410	1.356	1.453	1.254	1.370
**Lymphography**	3.104	3.203	5.110	4.633	4.436	**2.506**	5.390	4.084
**M-of-n**	0.403	0.403	0.403	0.404	0.404	0.401	0.389	**0.323**
**PenglungEW**	66.689	103.142	81.710	87.756	110.113	**62.330**	120.123	118.180
**SonarEW**	0.900	0.933	1.171	1.362	0.914	**0.614**	1.343	1.335
**SpectEW**	0.482	0.512	0.492	**0.474**	0.515	0.634	0.616	0.525
**CongressEW**	**2.628**	3.043	4.164	3.322	3.171	3.096	3.625	3.118
**IonosphereEW**	1.077	0.850	1.809	0.771	1.620	**1.023**	1.585	1.399
**KrvskpEW**	0.852	0.796	0.781	0.753	0.794	0.773	0.769	**0.744**
**Tic-tac-toe**	0.050	0.055	0.054	0.053	**0.047**	0.050	0.051	0.048
**Vote**	3.337	3.831	4.047	3.353	2.480	3.647	**2.219**	3.186
**WaveformEW**	6.178	5.903	6.110	6.314	5.823	5.875	5.485	**5.241**
**WineEW**	7.742	7.332	6.392	7.217	8.567	8.648	**6.126**	6.268
**Zoo**	299.533	**226.958**	288.376	267.946	260.639	265.226	244.569	298.357

#### Limitations

The main limitation of the methodology proposed in this paper is the non-exact repeatability of the optimization results. We observed that at different applications of the algorithm, the subset of features selected might differ. Although the resulting solutions are all good solutions, it may be confusing for the user to determine which subset to consider. The proposed algorithm works on the wrapper-based feature selection approach using the KNN classifier as a simple one. The running time may increase when switching to another classifier, such as support vector machine (SVM) or random forest (RF). Therefore, switching to a different classifier should be carefully handled, particularly if the algorithm is adopted in real-time applications.

## 4 Conclusions

In this paper, we address the feature selection problem by developing a chaos-based version of a recently proposed meta-heuristic algorithm, namely, antlion optimization (ALO). A parameter whose setting is crucial for the algorithm performance is adapted using chaos principles. The proposed chaotic antlion optimization (CALO) is applied to a common challenging optimization problem: feature selection in the wrapper mode. The feature selection is formulated as a multi-objective optimization task with a fitness function reflecting the classification performance and the reduction in the number of features. The proposed system is evaluated using 18 different datasets against a number of evaluation criteria. We developed this method with particular interest in datasets generated in biology, as these data typically have a large number of attributes and a low number of instances. CALO proves to be more efficient compared to ALO, PSO, and GA regarding the quality of the features selected. CALO is able to converge to the same optimal solution for a higher number of applications compared to ALO, PSO, and GA, regardless of the stochastic searching and the chaotic adaptation. The performance of CALO is better than that of the other methods over the test data considered.

## References

[1] Chuang LY, Tsai SW, Yang CH. Improved binary particle swarm optimization using catfish effect for feature selection. Expert Systems with Applications. 2011;38(10):12699–12707.

[2] Dash M, Liu H. Feature selection for Classification. Intelligent Data Analysis. 1997;1(3):131–156. 10.1016/S1088-467X(97)00008-5

[3] Huang CL. ACO-based hybrid classification system with feature subset selection and model parameters optimization. Neurocomputing. 2009;73(1–3):438–448. 10.1016/j.neucom.2009.07.014

[4] Duda RO, Hart PE, Stork DG. Pattern Classification. 2nd ed Wiley-Interscience; 2000.

[5] Chen Y, Miao D, R W. A rough set approach to feature selection based on ant colony optimization. Pattern Recognition Letters. 2010;31(3):226–233. 10.1016/j.patrec.2009.10.013

[6] Kohavi R, John GH. Wrappers for feature subset selection. Artificial Intelligence. 1997;97(1):273–324. 10.1016/S0004-3702(97)00043-X

[7] Xue B, Zhang M, Browne WN. Particle swarm optimisation for feature selection in classification: Novel initialisation and updating mechanisms. Applied Soft Computing. 2014;18:261–276. 10.1016/j.asoc.2013.09.018

[8] Guyon I, Elisseeff A. An introduction to variable and attribute selection. Machine Learning Research. 2003;3:1157–1182.

[9] Whitney A. A direct method of nonparametric measurement selection. IEEE Transactions on Computers. 1971;C-20(9):1100–1103. 10.1109/T-C.1971.223410

[10] Marill T, Green D. On the effectiveness of receptors in recognition systems. IEEE Transactions on Information Theory. 1963;9(1):11–17. 10.1109/TIT.1963.1057810

[11] Xue B, Zhang M, Browne WN. Particle swarm optimization for feature selection in classification: a multi-objective approach. IEEE transactions on cybernetics. 2013;43(6):1656–1671. 10.1109/TSMCB.2012.2227469 24273143

[12] Shoghian S, Kouzehgar M. A Comparison among Wolf Pack Search and Four other Optimization Algorithms. Computer, Electrical, Automation, Control and Information Engineering. 2012;6(12):1619–1624.

[13] Eiben AE, Raue PE, Ruttkay Z. Genetic algorithms with multi-parent recombination In: Parallel Problem Solving from Nature—PPSN III, International Conference on Evolutionary Computation. Springer; 1994 p. 78–87.

[14] Kennedy J, Eberhart R. Particle swarm optimization. IEEE International Conference on Neural Networks. 1995;4:1942–1948.

[15] Holland JH. Adaptation in natural and artificial systems. Cambridge, MA, USA:MIT Press; 1992.

[16] Eberhart R, Kennedy J. A New Optimizer Using Particle Swarm Theory In: International Symposium on Micro Machine and Human Science. IEEE; 1995 p. 39–43.

[17] Mirjalili S. The Ant Lion Optimizer. Advances in Engineering Software. 2015;83:80–98. 10.1016/j.advengsoft.2015.01.010

[18] Chen H, Jiang W, Li C, Li R. A Heuristic Feature Selection Approach for Text Categorization by Using Chaos Optimization and Genetic Algorithm. Mathematical Problems in Engineering. 2013;2013:1–6.

[19] Chakraborty B. Genetic algorithm with fuzzy fitness function for feature selection In: International Symposium on Industrial Electronics. IEEE; 2002 p.315–319.

[20] Chakraborty B. Feature subset selection by particle swarm optimization with fuzzy fitness function In: Third International Conference on Intelligent System and Knowledge Engineering. IEEE; 2008 p. 1038–1042.

[21] Neshatian K, Zhang M. Genetic programming for feature subset ranking in binary classification problems In: Genetic Programming. Berlin: Springer; 2002 p. 121–132.

[22] Gao HH, Yang HH, Y WX. Ant colony optimization based network intrusion feature selection and detection In: International Conference on Machine Learning and Cybernetics. IEEE; 2005 p. 3871–3875.

[23] Ming H. A rough set based hybrid method to feature selection In: International Symposium on Knowledge Acquisition and Modeling. IEEE; 2008 p. 585–588.

[24] Chuanwen J, Bompard E. A hybrid method of chaotic particle swarm optimization and linear interior for reactive power optimisation. Mathematics and Computers in Simulation. 2005;68(1):57–65. 10.1016/j.matcom.2004.10.003

[25] Kim H, Howland P, Park H. Dimension reduction in text classification with support vector machines. Journal of Machine Learning Research. 2005;6:37–53.

[26] Oh IS, Lee JS, Moon BR. Hybrid genetic algorithms for feature selection. IEEE Transactions on Pattern Analysis and Machine Intelligence. 2004;26(11):1424–1437. 10.1109/TPAMI.2004.105 15521491

[27] Landassuri-Moreno V, Marcial-Romero JR, Montes-Venegas A, Ramos MA. Chaotic Time Series Prediction with Feature Selection Evolution In: Conference in Electronics, Robotics and Automotive Mechanics. IEEE; 2011 p. 71–76.

[28] Gholipour A, Araabi BN, Lucas C. Predicting Chaotic Time Series Using Neural and Neurofuzzy Models: A Comparative Study. Neural Processing Letters. 2006;24(3):217–239. 10.1007/s11063-006-9021-x

[29] Vohra R, Patel B. An Efficient Chaos-Based Optimization Algorithm Approach for Cryptography. Communication Network Security. 2012;1(4):75–79.

[30] Ren B, Zhong W. Multi-objective optimization using chaos based PSO. Information Technology. 2011;10(10):1908–1916. 10.3923/itj.2011.1908.1916

[31] Raouf OA, Baset MA, henawy IE. An Improved Chaotic Bat Algorithm for Solving Integer Programming Problems. Modern Education and Computer Science. 2014;6(8):18–24. 10.5815/ijmecs.2014.08.03

[32] Aguirregabiria JM. Robust chaos with variable Lyapunov exponent in smooth one-dimensional maps. Chaos Solitons & Fractals. 2014;42(4):2531–2539. 10.1016/j.chaos.2009.03.196

[33] Saremi S, Mirjalili S, Lewis A. Biogeography-based optimisation with chaos. Neural Computing and Applications. 2014;25(5):1077–1097. 10.1007/s00521-014-1597-x

[34] Yang XS. Nature-Inspired, Metaheuristic Algorithms. 2nd ed United Kingdom: Luniver Press; 2010.

[35] Vieira SM, Sousa JMC, Runkler TA. Two cooperative ant colonies for feature selection using fuzzy models. Expert Systems with Applications. 2010;37(4):2714–2723. 10.1016/j.eswa.2009.08.026

[36] Bache K, Lichman M. UCI Machine Learning Repository. type. 2013 [cited 2016 Jan 15];Available from: http://archive.ics.uci.edu/ml.

[37] Raman B, Ioerger TR. Instance Based Filter for Feature Selection. Machine Learning Research. 2002;1:1–23.

